# Triboelectric charging of melt-blown nonwoven filters with high filtration efficiency

**DOI:** 10.1038/s41598-022-04838-3

**Published:** 2022-01-21

**Authors:** Hong Wang, Yanjin Wu, Jiang Wang

**Affiliations:** Key Laboratory of Textile Science and Technology, Ministry of Education, Donghua University, No. 2999 North Renmin Road, Songjiang District, Shanghai, 201620 China

**Keywords:** Engineering, Materials science

## Abstract

As a novel technology to convert low-frequency energy into electric power, the triboelectric nanogenerator is a hot research topic recently. However, the nature of charge carriers and their transfer mechanisms still remain poorly understood, especially for the cases of liquid–solid triboelectric nanogenerator. In this paper, charges produced by a triboelectric charging process were designed to provide melt-blown nonwoven fabrics with high filtration efficiency by making full use of the electrostatic attraction filtration mechanism. Influences of water conductivity and drying temperature on the filtration efficiency of melt-blown nonwoven fabrics were investigated. And the corresponding properties such as the surface charge potential and charge stability were analyzed by using the electrostatic voltmeter, bio atomic force microscope and thermally stimulated discharge technique. In addition, metal and inorganic elements in the masterbatch and water before and after triboelectric charging were measured in order to uncover the charge transfer mechanism. Melt-blown nonwoven fabrics with filtration efficiency as high as 96.8% was obtained through the triboelectric charging treatment by using water with the conductivity as low as 1.1 μS/cm for the first time. Negative and positive surface charge density appeared randomly on both sides of melt-blown nonwoven fabrics after the triboelectric charging treatment from the bio atomic force microscope measurement while only one kind of surface charge density can be achieved in the research of TENG, that is, negative or positive. It seems there are both electron and ion transfers during the triboelectric charging process and electron transfer seems to have more important contribution for the generation of charges.

## Introduction

As the functional middle layer of face masks, the meltblown nonwoven fabric (MNF) can capture particles according to inertial collision, interception, and diffusion filtration mechanisms^1^. Except for filtration efficiency, respiratory resistance of face masks is another factor concerned by consumers which has big influence on the wearing comfort, while there is an intrinsic conflict between them for conventional face masks.

As we know, electret filtration materials are able to improve the ability to capture particles without increasing the pressure drop by making full use of the electrostatic attraction filtration mechanism. To achieve this purpose, MNF is usually corona charging treated just by passing through a high voltage electric field continuously after the polymer melt is extruded out of the spinneret and thermally self-bonded into a fibrous web^2^. To further improve the charge density and stability, additives such as SiO_2_, BaTiO_3_, boehmite or boron nitride nanosheets are usually added into the polymer melt^3–5^. One of the disadvantages of the corona charging process is the oxidation that occurs due to highly energetic charge carriers. This increases the hydrophilicity of fibers, which results in the adsorption of a water film which is susceptible to a gradual degradation of surface charge, eventually exhibiting surface conductivity and charge decay^6^.

Besides the corona charging method, triboelectric charging has been developed by 3 M Company as another mysterious technique which is similar to the solid–liquid contact electrification process^7,8^. In 1999, a paper concerned the efficiency of electrostatic charging of water-sprayed fibers during the melt-blowing process was published. It was found that the charging efficiency was not affected by the water-spraying process if the amount of spraying was controlled^9^. In 2014, a paper in Korean was published about the melt-blown nonwoven filter for medical masks manufactured using an online triboelectric charging system^10^. The effects of the basis weight of MNF and die to collector distance on the filtration performance of MNF were investigated while there was no deep discussion about the charge origin in the triboelectric charging process. In addition to the above research, few study can be found about the triboelectric charging of fabrics in the literature.

Since the outbreak of COVID-19 at the beginning of 2020, face masks have attracted great attention worldwide and hydro charging technique has been widely used to produce MNF with higher filtration efficiency and long-term stability as the key layer of face masks while remain the same pressure drop, although the underlying mechanism is still not yet understood.

In addition, since invented in 2012, much research is now focused on triboelectric nanogenerator (TENG) which exhibits potential application as a power source, self powered sensor, capacitor and blue energy harvester^11–15^. Among them, TENG based on liquid–solid triboelectrification is proposed with new strategies toward harnessing water kinetic energy^16–18^. Although triboelectrification is a well known phenomenon for more than 2600 years, its scientific understanding is still under debate, especially for the cases of liquid–solid contact electrification^19^.

Based on the above background and our previous study, in this paper, MNF with high filtration efficiency was prepared in our lab by using a simulating triboelectric charging equipment. The corresponding properties such as the surface charge potential and charge stability were analyzed. And metal and inorganic elements in the masterbatch and water before and after triboelectric charging treatment were measured with the aim to uncover the charge transfer mechanism of the triboelectric charging treatment which is helpful to explore the mechanism of water–solid TENG as well.

## Experimental

### Triboelectric charging experiment

**Figure 1:**
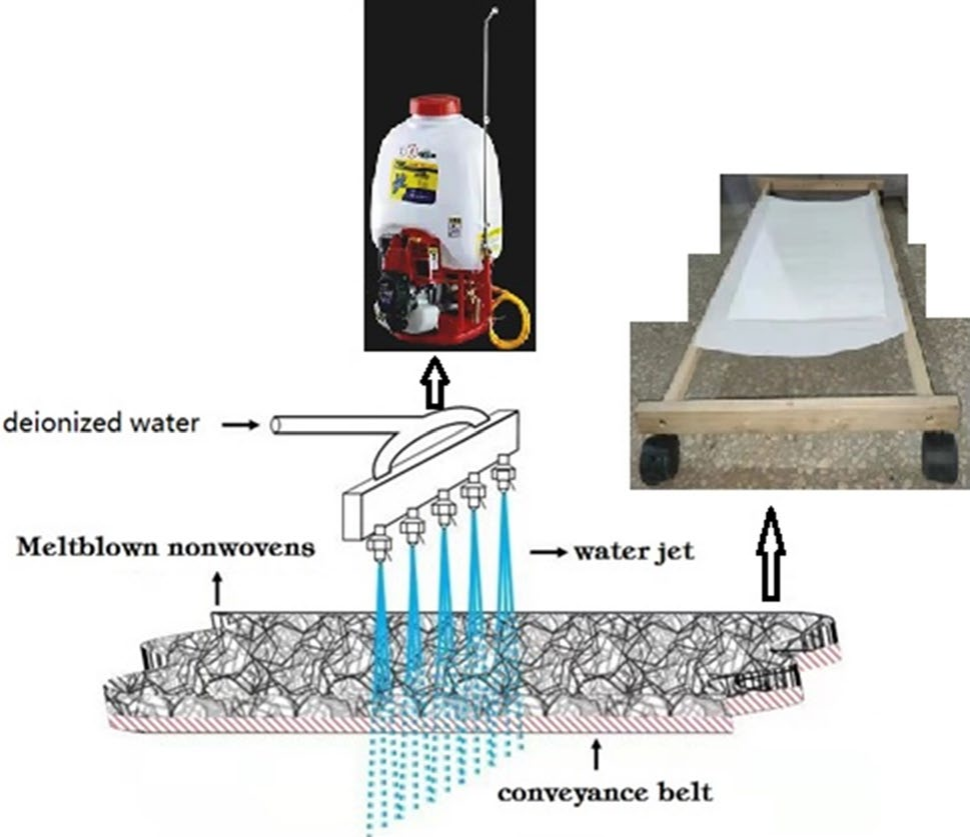
The schematic diagram of triboelectric charging process.

**Figure 2:**
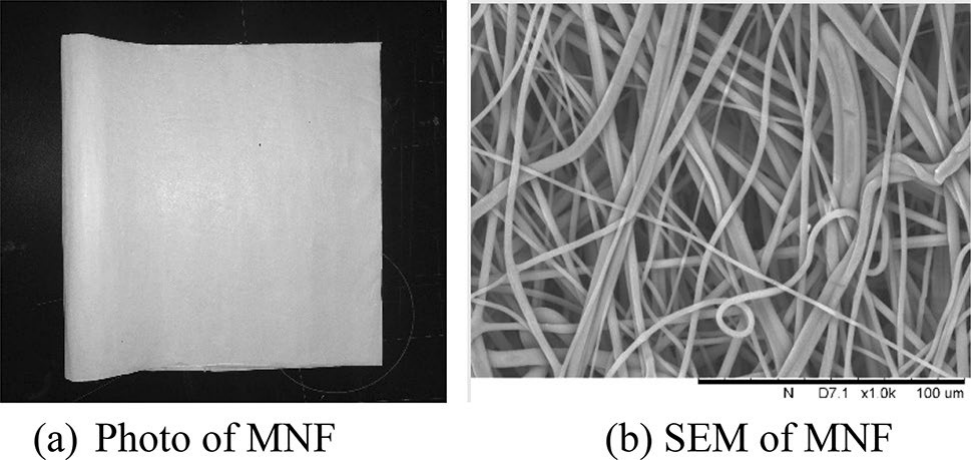
Images of triboelectric charging treated MNF.

In this paper, a simulating triboelectric charging equipment was set up in our lab with a portable petrol powered sprayer (the water pressure is in the range of 1 ~ 3 MPa) to generate water jets with desired speed, as illustrated in Fig. 1. Deionized water with different conductivity was sprayed out of the nozzle of the portable sprayer and continuously impinged on up and down sides of MNF repeatedly for 1 min, which was put on a shelf with wheels and could be moved forward and back manually.Then the wet MNF was dried in an oven for 2 min under certain temperature, as shown in Fig. 2. MNF used in the experiment was composited with 2.5% electret masterbatch which was added during the melt-blowing process of polypropylene, with the basis weight of 25 g/m^2^.

### Characterization

The conductivity of the water used for the triboelectric charging experiment was measured by using a conductometer (FE30K, FiveEasy Plus).

The filtration efficiency of MNF with fiber diameter of 3.0 µm was conducted by using an automated filter tester (TSI 8130, TSI Inc., USA). The concentration of Dioctyl Phthalate Particles (DOP) is in a range of 50–200 mg/m^2^, the Mass Median Diameter (MMD) < 0.3 μm, Count Median Diameter (CMD) < 0.075 μm, the flow rate: 95 L/min.

The average pore size of MNF was measured by a bubble-point test utilizing a capillary flow porometer (CFP‐1100AI, Porous Materials Inc., USA).

An electrostatic voltmeter (Model 542, TREK Inc., USA) was used to determine the surface charge potential of MNF with the size of 12 cm × 12 cm which was laid on an insulating desk. The values of surface charge potential at different areas on the MNF were recorded one by one when the sensor hold by hand was moving over the MNF 15 mm away. In order to reflect the charge potential distribution, each sample was tested for three times, with 30 points were recorded on the front side and back side, respectively.

The Dimension FastScan Bio Atomic Force Microscope (Bruker Co., USA) was used to measure the surface potential of MNF before and after triboelectric charging treatment. The model of the conducting probe was OMCL-AC240TM-R3 with the spring constant and resonance range of 2 N/m and 2 V, respectively. The lift-off distance of the probe was 200 nm.

The open circuit thermally stimulated discharge (TSD) measurements of MNF were carried out in a system consisting of a temperature controlled oven with a linear heating rate of 3℃/min, an electrometer (Model6514, Keithley), and a data processing computer. The linear temperature range of the TSD test was 30 − 150℃.

Metal and inorganic elements in the electret masterbatch and water before and after triboelectric charging treatment were measured by using inductively coupled plasma analysis (Prodigy, Leeman, America) with argon as the carrier gas.

## Experimental results

### The influence of water conductivity on the filtration efficiency of MNF

**Figure 3:**
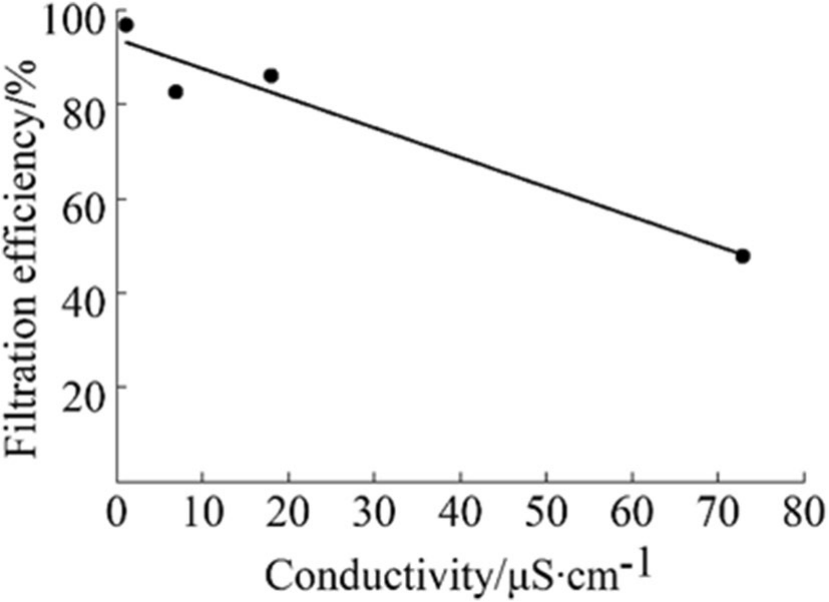
The influence of water conductivity on the filtration efficiency of MNF.

The electrical conductivity of water is used in many industries as an indication of the purity of water. The conductivity of tap water is in the range of 125 ~ 1250 μS/cm while the conductivity of distilled water used widely in the study of solid–liquid contact electrification is only 0.18μS/cm (the resistivity is 18.2 MΩ·cm)^20^. As we know, excessive purification of water will increase the cost of production unnecessarily. Hence, in this paper, the influence of the electrical conductivity of water on the effect of triboelectric charging was studied at first. MNF were triboelectric charging treated by using water with the electrical conductivity of 1.1, 6.9, 18.0 and 72.9 μS/cm, respectively, while keeping other experimental conditions the same. Effects of the electrical conductivity of water on the filtration efficiency of the obtained MNF were shown in Table 1 and Fig. 3. In addition, in order to rule out the influence of physical filtration effects, the mean pore size of MNF before and after triboelectric charging treatment were measured as well, as shown in Table 1.

**Table 1:** Effects of the electrical conductivity of water on the filtration efficiency and mean pore size of MNF.

samples	Conductivity (μS/cm)	Filtration efficiency (%)	Mean pore size (μm)
1	1.1	96.8	9.7
2	6.9	82.5	10.2
3	18.0	86.0	10.3
4	72.9	47.8	9.9

It can be found from Table 1 and Fig. 3 that the filtration efficiency of MNF decreased with the increase of the conductivity of water significantly. As we know, the pore size of filters has a great influence on the filtration efficiency according to the physical filtration mechanism. However, as presented in the Table 1, the mean pore size of triboelectric charging treated MNF by using water with different conductivity did not change obviously. It is believed that the variation was mainly due to the compression being employed during the triboelectric charging process. Therefore, it is believed that static charges on fiber surface of MNF generated after the triboelectric charging treatment have important contribution to the filtration efficiency of MNF by making use of the electrostatic attraction filtration mechanism, consistent with the findings in the study of solid–liquid TENG which is reported that solutes in the solution, pH value of the solution and the hydrophilicity of the solid affect the ratio of electron transfers to ion transfers^21^.

Based on the coupling between the triboelectric effect and electrostatic induction, TENG has proven to be a cost-effective and environmentally friendly technology for mechanical energy harvesting, self-powered devices and systems, and air filter. A triboelectric air filter consisting of five layers of the polytetrafluoroethylene (PTFE) and nylon fabrics can be charged by simply rubbing the PTFE and nylon fabrics against each other. After charging, the air filter has a removal efficiency of 84.7% for PM0.5, 96.0% for PM2.5^22^. A face mask based on the Poly(vinylidene fluoride) electrospun nanofiber film and TENG driven by respiration is developed. The face mask can continually provide electrostatic charges by respiration and shows a removal efficiency of coarse and fine particulates as high as 99.2wt%, and its removal efficiency of ultrafine particulates is as high as 86.9wt%^23^. In this study, MNF with filtration efficiency of NaCl particles with diameter of 0.3 μm is as high as 96.8% through the triboelectric charging treatment by using water with the conductivity as low as 1.1 μS/cm, much higher than all the above reports.

### The influence of drying temperature on the filtration efficiency of MNF

In order to understand influences of the drying temperature on the filtration efficiency of MNF, the wet MNF was dried in an oven under 110℃, 130℃, 150℃ and 160℃ respectively, after the triboelectric charging treatment by using deionized water with the conductivity of 1.1μS/cm, and the results were shown in Fig. 3. In comparison, the filtration efficiency of triboelectric charging treated MNF dried under room temperature (25℃) was measured as well.

**Figure 4:**
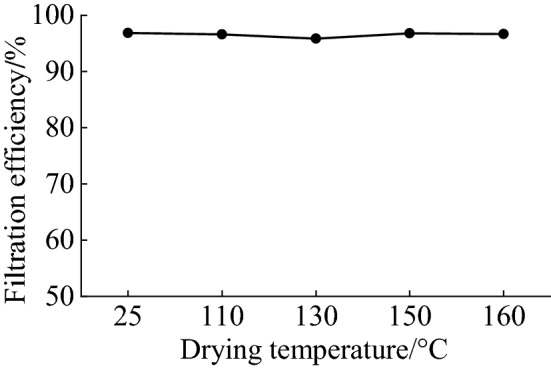
The influence of drying temperature on the filtration efficiency of MNF.

It can be found from Fig. 4 that triboelectric charged MNF dried under different temperature exhibited similar filtration efficiency, suggesting that the drying temperature does not influence the filtration efficiency of MNF, and static charges on fiber surface of MNF generated after the triboelectric charging treatment have good thermal stability as well. On the other hand, it was found that MNF dried under 160℃ shrank obviously, suggesting that the drying temperature should not be higher than 150℃.

### Surface charge potential of MNF

**Figure 5:**
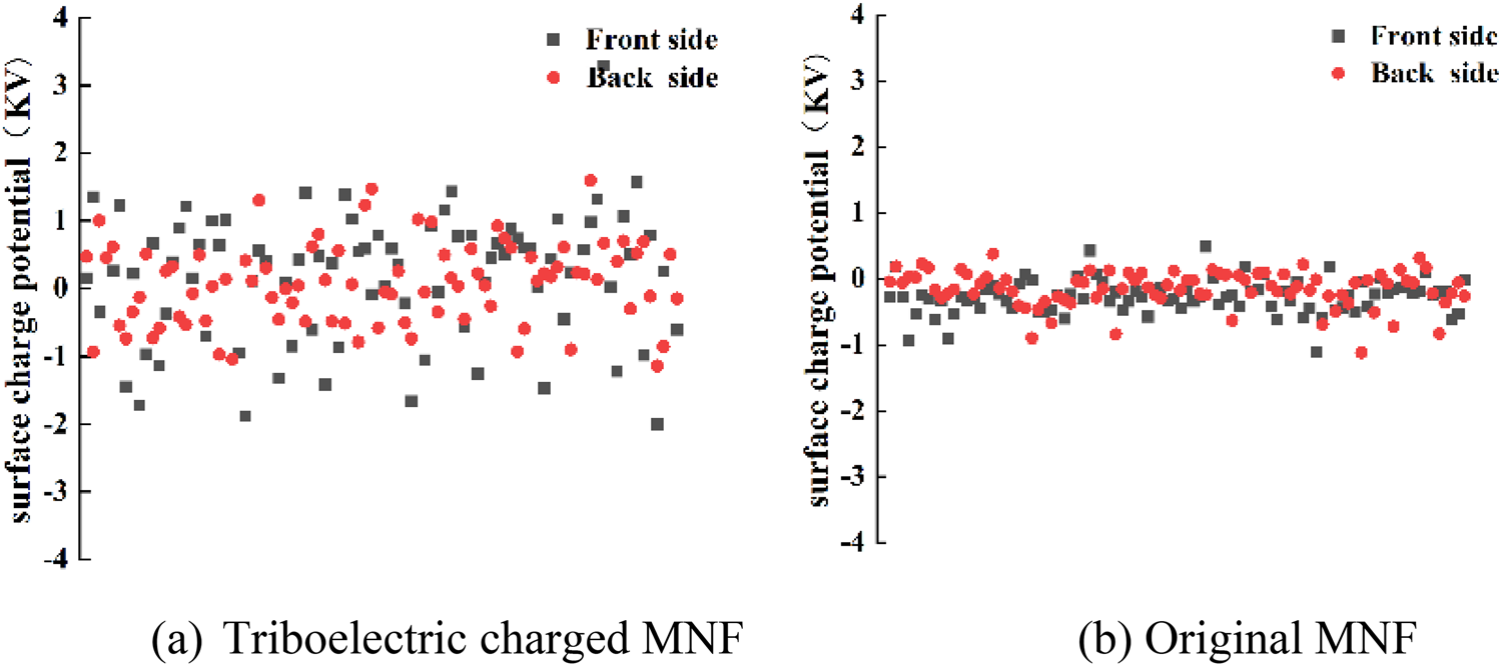
Surface charge potential of MNF before and after triboelectric charging treatment.

In order to verify if the filtration efficiency improvement of triboelectric charged MNF is due to the extra electrostatic charges obtained by the triboelectric charging treatment, the surface electrostatic potential of triboelectric charged MNF (sample 1 in Table 1) and the original MNF were measured by using a non-contacting voltmeter and the results were shown in Fig. 3. In order to reflect the charge distribution, each sample was tested for 90 times and the corresponding surface charge potential distribution diagram was performed by plotting the surface charge potential as the vertical y-axis against the sequence number of each surface charge potential as the horizontal x-axis, as shown in Fig. 5.

As shown in Fig. 5, the surface charge potential of triboelectric charged MNF was much higher than that of the original MNF, suggesting that the triboelectric charging treatment could generate charges on the fiber surface of MNF, resulting in the higher filtration efficiency consequently. Negative and positive charges appeared randomly on both sides of MNF treated by triboelectric charging process as shown in Fig. 5a while only one kind of surface charge density can be achieved in the research of TENG, that is, negative or positive^24^. In addition, it is believed that the surface charge potential of the original MNF is due to the polarization of additives and impurities in the polymer melt during the spinning and drawing process^25^.

**Figure 6:**
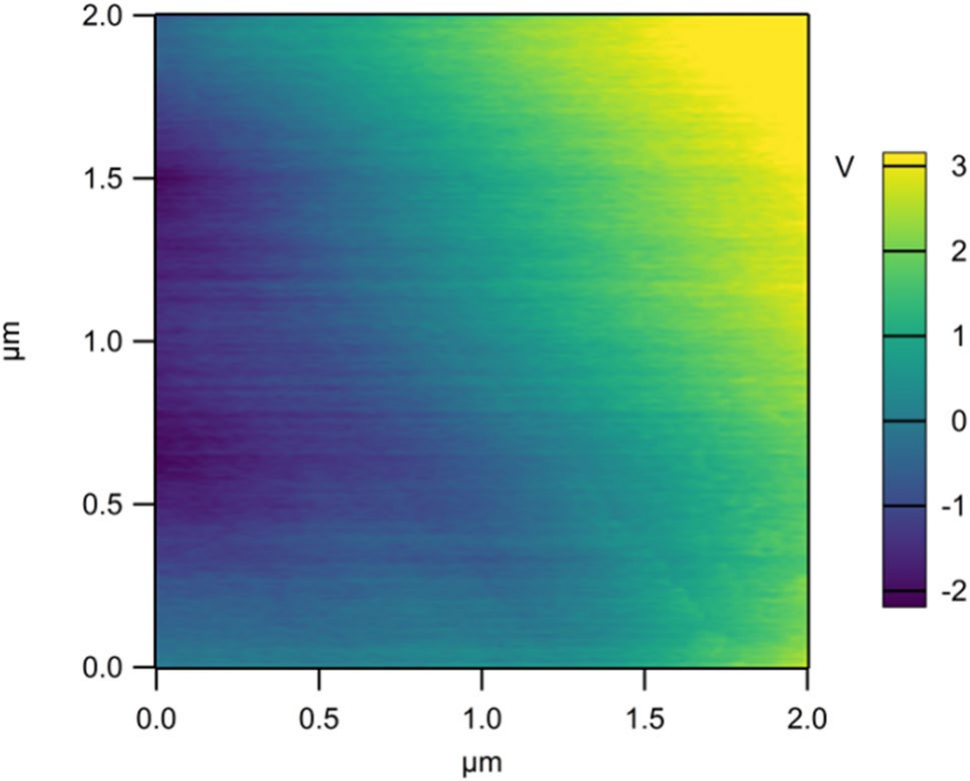
Surface charge density distribution of MNF treated by triboelectric charging.

To double check the nature of charges on MNF, the surface charge densities of sample 1 listed in Table 1 was measured by using a FastScan Bio Atomic Force Microscope (AFM) and the results were shown in Fig. 6.

It can be found from Fig. 6 that negative and positive charges appeared simultaneously on triboelectric charged MNF. To the best of our knowledge, the mean fiber diameter of MNF is in the range of 1 ~ 4 μm. Hence it can be concluded from Fig. 4 that a single fiber of MNF can be negatively and positively charged simultaneously during the triboelectric charging process, which is quite different from the charges widely reported in the study of TENG.

### Charge storage stability of triboelectric charged MNF

**Figure 7:**
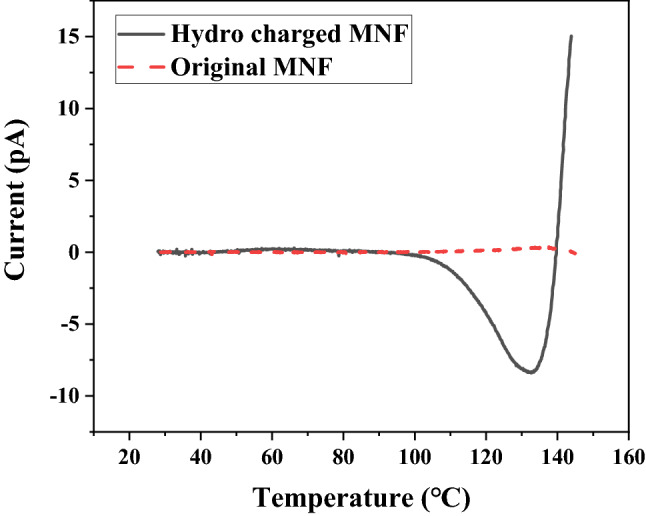
The TSD spectra of MNF before and after triboelectric charging treatment.

The thermally stimulated discharge (TSD) current measurement is often applied to study the stable behavior of charge storage^26^, which is helpful to uncover the nature of charge carriers and their transfer mechanism to some extent. In this paper, the charge stability of MNF before and after triboelectric charged treatment was investigated by TSD technique, as shown in Fig. 7.

It is reported that the medium-temperature peak between 100 and 130℃ on TSD spectra of charged polypropylene is attributed to the release of both inherent and injected charge carriers^27^. As demonstrated in Fig. 7, a peak at about 130℃appeared obviously on the TSD current spectra of triboelectric charged MNF, while the discharged current of virgin MNF over the entire temperature range was negligible, suggesting that a great amount of charges were injected into MNF by triboelectric charging treatment, which is consistent with the surface charge potential analysis as shown in Fig.6.

### Composition analysis of the triboelectric charging masterbatch and water before and after triboelectric charging treatment

As we know, only MNF added with functional masterbatch can achieve higher filtration efficiency by triboelectric charging treatments. Hence it is necessary to study the composition and function of the functional masterbatch which might be helpful to identity charge carriers (electrons or/and ions) and further understand the mechanisms underlying the triboelectric charging process. In this paper, metal and inorganic elements of the masterbatch used in the experiment were measured by using ICP and the results were shown in Table 2. In comparison, a commercial corona charging masterbatch was measured as well.

**Table 2:** metal and inorganic elements of triboelectric charging and corona charging masterbatches.

Content (mg/g)	Mg	Ba	Ca	Si	B	Al	K	Na
Triboelectric	0.08	1.37	1.17	0.15	1.77	0.43	1.59	0.29
Corona	2.83	≤ 0.01	1.02	0.35	≤ 0.01	0.08	0.09	0.61

It can be found from Table 2 that the metal and inorganic elements content of the triboelectric charging masterbatch was quite different from that of corona charging masterbatch. Mg content of the triboelectric charging masterbatch was much lower than that of the corona charging masterbatch while there were more Ba and B in it. In addition, the content of Ca was similar in the two kinds of masterbatches, which might help to improve the charge stability of MNF.

In the corona charging process, meltblown nonwoven fabrics are often prepared by blending with dielectric additives such as SiO_2_, Al_2_O_3_, BaTiO_3_, boehmite, and boron nitride nanosheets, which will develop a quasi-permanent polarized electrical charge when they are subjected to a high voltage electric field. In addition, it is believed that the charge traps mainly originate from the interfaces between crystallite and amorphous region of MNF. As a kind of nucleating agent, MgSt can improve the crystallinity of MNF with more fine-grained crystallite. Hence it is often added as the charge enhancer as well^28,29^.

However, it is impossible for the triboelectric charging masterbatch to polarize electrically because the triboelectric charging treatment doesn’t operate necessarily in a high voltage electric field. Hence, it is believed that the masterbatch used in this paper is able to lose and/or receive electrons from the water spray during the triboelectric charging process.

In order to verify whether the ion transfer also appears during the triboelectric charging process as those described in the research of TENG, metal and inorganic elements in the water before and after triboelectric charging treatment were examined by using ICP which is a promising technique for analysis of trace elements in solutions and has been rated as the most powerful tool in analytical chemistry. Metal and inorganic elements in the water before and after triboelectric charging process were shown in Table 3.

**Table 3:** Metal and inorganic elements in the water before and after triboelectric charging process.

(mg/L)	B	Ba	Ca	K	Si	Na
Original water	1.12	≤ 0.01	≤ 0.01	≤ 0.01	1.87	≤ 0.01
Triboelectric charged water	0.39	≤ 0.01	≤ 0.01	0.03	0.63	0.01

It can be found from Table 3 that the metal and inorganic elements in the triboelectric charged water were not higher obviously than those in the original water, suggesting that metal and inorganic elements on the fiber surface of MNF didn’t dissolve in the water during the triboelectric charging process. The content of B and Si in the original water was much higher than we expected because the apparatus for producing deionized water can’t remove them from the tap water. It is believed that electron transfer between the water jet and fibers of MNF is the main phenomenon during the triboelectric charging process. In other words, ion transfer between the MNF and water is not the main origin of static charges on fiber surface.

### The charge transfer mechanism of triboelectric charging process

Although the contact electrification phenomenon has been known since antiquity, the nature of charge carriers and their transfer mechanisms still remain poorly understood, especially for the cases of liquid–solid contact electrification. In the past few decades, at least three mechanisms have been proposed: electron transfer, ion transfer and material transfer^25^. On the other hand, other studies proposed that ions, instead of electrons, are transferred during contact electrification^30^. In addition, Pandey and co-workers studied the charge transferred and the amount of material transferred during the contact electrification by varying the degree of softness of a polymer and believe that material transfer seems to have an important contribution for the generation of charge by contact, especially for materials that are soft^31^. As the developer of TENG, Wang and co-workers proposed a Wang’s hybrid layer model to explain the solid–liquid contact electrification^24^. In Wang’s hybrid layer model, the water is assumed to be neutral before it contact with the solid. The water droplet gently separated from the grounded needle and instantly came into contact with the polymer surface in the drop-TENG experiment. However, in other study, it is reported that the water movement and impact can lead to an extensive accumulation of electrical charge in water due to the charge separation of water^16,32^.

**Figure 8:**
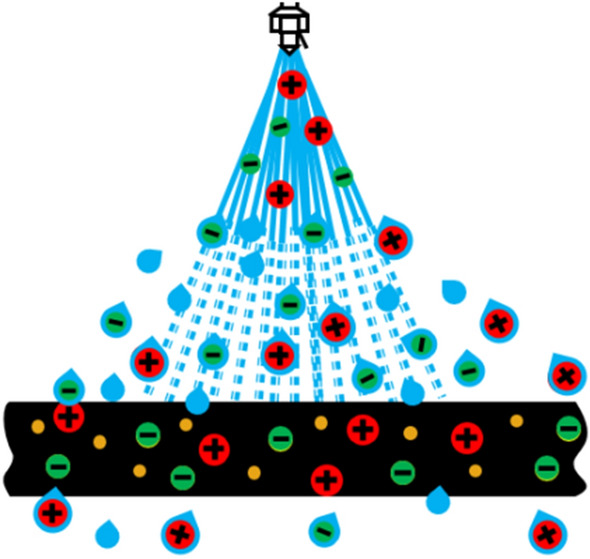
Diagram of charge transfer mechanism in the triboelectric charging process.

Based on the above experimental results and research on charge transfer mechanisms of liquid–solid contact electrification, a diagram of charge transfer mechanism of the triboelectric charging process is established, as illustrated in Fig. 8, and a charge transfer mechanism is proposed as follows.

Similar to the solid–liquid TENG, in the triboelectric charging process, it is believed that the water has been positively charged due to the lost of electrons caused by the friction with the pipeline. After spraying out of the nozzle, more charge separation happens due to the splashing and bubbling movement. Therefore, water droplets become positively, negatively, or neutrally charged before they contact with MNF. When positively and negatively charged water droplets come into contact with MNF, they will be neutralized after receiving electrons or transferring electrons to fibers of MNF. At the same time, neutral droplets will lose the lone pair electrons from the oxygen atoms caused by the contact electrification between water and fiber surface. Correspondingly, fibers of MNF will be charged positively and negatively as shown in Fig. 6, and the negative and positive surface potential of MNF is distributed randomly as shown in Fig. 5 as well.

It is believed that electron transfer between the water jet and fibers of MNF is the main phenomenon during the triboelectric charging process. In addition, with functional materbatch added, electron transfer between the water droplet and fiber surface become more efficiently and more available charge densities are filled, resulting in MNF with higher static surface potential consequently.

For producing electret filters, triboelectrification generally needs more than one components to be present in order to make full use of the electrostatic attraction filtration mechanism. That is, one kind of fibers from the more negative side of the triboelectric series, and one from the more positive side. However, MNF used for the hydro charging treatment is composed with only one kind of fibers which was blended with 2.5% electret masterbatch.

**Figure 9:**
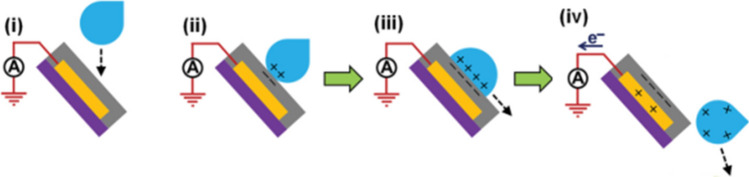
Working mechanism of the solid–liquid TENG.

In the study of the solid–liquid TENG^19^, as shown in Fig. 9, when a water drop falls down and contacts with the thin film, the ionization of surface groups on the film will cause it to be negatively charged and cause a positively charged electrical double layer (EDL) on the contact surface of the water drop to maintain electrical neutrality. As the water drop is leaving the thin film, a negative electric potential difference will be established between the electrode and ground. In the short-circuit case, electrons are transferred from the electrode to ground.

**Figure 10:**
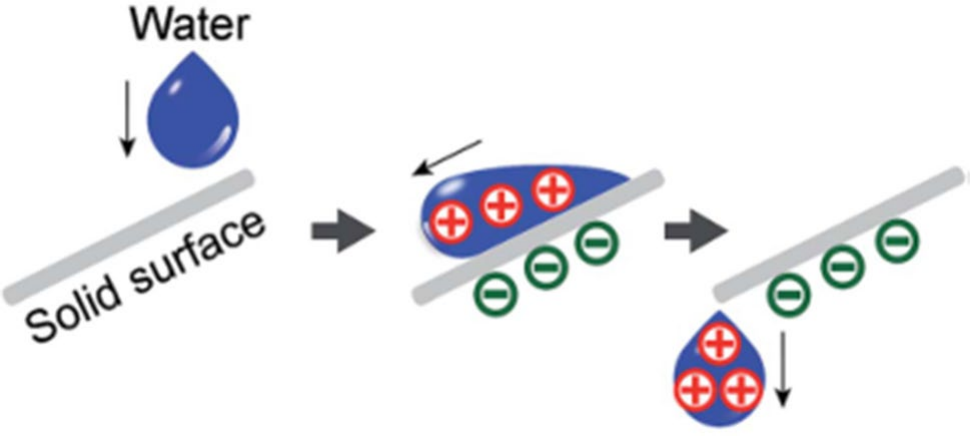
Working mechanism of the solid–liquid electrification.

In the study of solid–liquid electrification^33^, as shown in Fig. 10, when water (initially uncharged) flows across, and then falls off a solid surface under its own weight, it acquires a net positive charge. Consequently, the solid acquires a net negative charge.

In conclusion, solid with different charge property is obtained after the solid–liquid electrification/TENG and triboelectric charging treatment studied in this paper. The solid surface is negatively charged in the solid–liquid electrification and solid–liquid TENG while fibers of MNF are charged positively and negatively randomly after the triboelectric charging experiment.

## Conclusion

In conclusion, a meltblown nonwoven filter with high filtration efficiency was developed by triboelectric charging process and the charge origin mechanism was analyzed for the first time. The effect of the water conductivity and drying temperature on the filtration efficiency of MNF was investigated. In addition, metal and inorganic elements in the masterbatch and water before and after triboelectric charging treatment were measured in order to uncover the charge transfer mechanism. More details are summarized as follows:

The conductivity of water played a significant role on the filtration efficiency of triboelectric charged MNF which decreased with the increase of the conductivity of water significantly. MNF with filtration efficiency as high as 96.8% was obtained by using water with the conductivity as low as 1.1 μS/cm. On the other hand, the drying temperature didn’t have obvious influence on the filtration efficiency of triboelectric charged MNF.

The metal and inorganic elements content of the masterbatch used in this paper was quite different from the corona charging masterbatch, in which the Mg content was much lower than that of the corona charging masterbatch while more Ba and B elements appeared. On other hand, metal and inorganic elements in the triboelectric charged water were not higher obviously than those in the original water, suggesting that ion transfer between the MNF and water is not the main origin of static charges on fiber surface.

It is found that negative and positive charges generated simultaneously on fiber surface of MNF after triboelectric charging treatment which is more desirable as a filter. In contrast, only one kind of surface charges can be achieved in the study of solid–liquid TENG. It is believed that electron transfer between the water jet and fibers of MNF during the triboelectric charging process is the main origin of charge carriers, rather than originating from ions transfer.

## Supplementary Information

Supplementary Information.

## Data Availability

All data generated or analysed during this study are included in this published article and its supplementary information file.
