# Supplementary material for: Triboelectric charging of melt-blown nonwoven filters with high filtration efficiency
Source: Sci Rep. 2022 Jan 21;12:1146. doi: 10.1038/s41598-022-04838-3 (PMC8782902; doi:10.1038/s41598-022-04838-3)
Supplement: Supplementary file 1 — Supplementary Information. [file 41598_2022_4838_MOESM1_ESM.docx]

**Supplementary Information for:**

**“Triboelectric charging of melt-blown nonwoven filters with high filtration efficiency”**

**Hong Wang, Yanjin Wu, Jiang Wang**

An experiment is designed to measure the surface charge potential of MNF in different layer. A thin layer of triboelectric charged MNF was peeled off gently. After measuring the surface charge potential, a thin layer was peeled off once again and the surface potential was measured as well. The surface charge potential of triboelectric charged MNF at different layer was shown in Fig.S1. The second layer and third layer refer to the original sample which was peeled off once and two times, respectively.


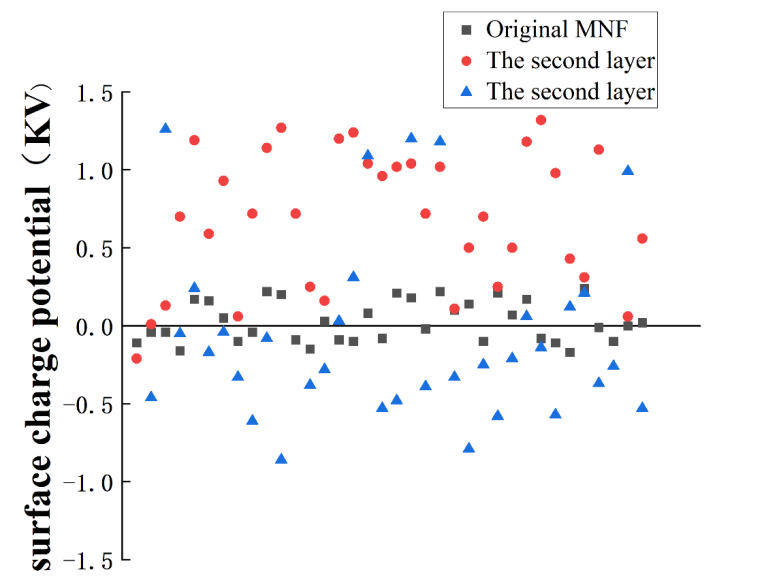


Fig.S1. Distribution diagram of the surface charge potential of triboelectric charged MNF at different layer

It can be seen from Fig.S1 that the surface charge potential of triboelectric charged MNF at different layer was totally different, suggesting charges were distributed randomly in each layer. In other words, negative and positive charges appeared on the fiber surface of MNF after triboelectric charging treatment and distributed randomly, which is helpful to improve the filtration efficiency of MNF. On the other hand, the surface potential of MNF can’t reflect the net charges of MNF and can’t be used directly to evaluate the filtration efficiency directly.
